# Nursing Support for Nausea and Vomiting in Patients With Cancer: A Scoping Review

**DOI:** 10.7759/cureus.48212

**Published:** 2023-11-03

**Authors:** Masamitsu Kobayashi, Kohei Kajiwara, Miharu Morikawa, Yusuke Kanno, Kimiko Nakano, Yoshinobu Matsuda, Yoichi Shimizu, Taichi Shimazu, Jun Kako

**Affiliations:** 1 Graduate School of Nursing Science, St. Luke’s International University, Tokyo, JPN; 2 Faculty of Nursing, Japanese Red Cross Kyushu International College of Nursing, Munakata, JPN; 3 Graduate School of Medicine, Kyoto University, Kyoto, JPN; 4 Nursing Science, Tokyo Medical and Dental University, Tokyo, JPN; 5 Clinical Research Center for Developmental Therapeutics, Tokushima University Hospital, Tokushima, JPN; 6 Department of Psychosomatic Internal Medicine, National Hospital Organization Kinki-Chuo Chest Medical Center, Sakai, JPN; 7 Faculty of Nursing, National College of Nursing, Tokyo, JPN; 8 Division of Behavioral Sciences, National Cancer Center Institute for Cancer Control, National Cancer Center, Tokyo, JPN; 9 Department of Nursing, Graduate School of Medicine, Mie University, Mie, JPN

**Keywords:** scoping review, terminally ill, quality of life, psychological support, cancer disease symptoms, nursing support, nausea and vomiting, cancer

## Abstract

Nausea and vomiting are symptoms commonly experienced by patients with advanced cancer and have a wide range of causes, including pharmacological interventions. Additionally, multiple factors often simultaneously cause nausea and vomiting. These highly distressing symptoms may be directly or indirectly related to the disease and can significantly impact both the physical and psychological well-being of patients. This study aims to identify the nursing support provided to reduce nausea and vomiting experienced by patients with cancer. This study followed the Preferred Reporting Items for Systematic Reviews and Meta-Analyses Extension for Scoping Reviews checklist and Arksey and O’Malley’s framework. We searched the PubMed, the Cumulative Index to Nursing and Allied Health Literature, the Cochrane Central Register of Controlled Trials in the Cochrane Library, and the Ichushi-Web of the Japan Medical Abstract Society databases for all content published from the inception of each database through July 31, 2023. A total of 4,625 scientific articles were identified after literature screening. In total, 58 articles were included for full-text review, and 10 articles were finally selected for review. The types of study designs comprised six randomized controlled trials, three prospective observational studies, and one before-after study with no controls. The types of cancers included in the articles were colorectal, breast, lung, pancreatic, gynecological, stomach, and sarcoma. The total sample size of the study population was 793 patients (range = 12-281) for intervention studies and 4,333 patients (range = 20-4,197) for observational studies. Nursing support, extracted from the 10 articles, was classified into the following six types: massage therapy, acupressure, early palliative care, psychosocial support, self-symptom monitoring, and coordinated care. The review yielded six classifications of nursing support for nausea and vomiting in cancer patients. Future research should examine the feasibility of providing nursing support for nausea and vomiting in cancer patients.

## Introduction and background

Nausea is an entirely subjective experience, defined as “the sensation (or sensations) that immediately precede vomiting” [1]. Patients often state that they feel like they are about to vomit, feel “nauseous,” or have an “upset stomach.” Vomiting is a highly specific physical event, defined as “the rapid, forceful evacuation of gastric content in a retrograde fashion from the stomach up to and out of the mouth” [1]. Nausea and vomiting are symptoms commonly experienced by patients with advanced cancer [2,3] and have a wide range of causes, including pharmacological therapy, such as anticancer drugs and opioids; radiotherapy; abnormal, decreased, or enhanced gastrointestinal motility; and central nervous or psychological causes [4,5]. Additionally, multiple factors often simultaneously cause nausea and vomiting [1]. These highly distressing symptoms can be directly or indirectly related to the disease and can significantly impact patients’ physical and psychological well-being [6].

The pathophysiology of nausea and vomiting is straightforward and is thought to involve mostly lower brain structures without general involvement of the cerebral cortex or other areas of higher development. Nausea and vomiting are a reflex triggered by toxic substances, such as chemotherapeutic agents, within the body [7]. However, in the case of patients with advanced cancer, identifying and treating the cause of the disease is often difficult or even impossible; hence, patient care is focused on providing symptomatic treatment. Today, we have a multitude of options available, targeting various pathways, such as 5-HT3 receptor antagonists, NK1 receptor antagonists, corticosteroids, anxiolytics and antipsychotics, and even cannabinoids [8]. Therefore, antiemetics are recommended as the first choice of treatment for nausea and vomiting in patients with cancer [9-11]; however, concurrently incorporating non-pharmacological support is thought to be useful in relieving distressing symptoms [9,12]. Previous studies have focused on non-pharmacological support for treatment-related nausea and vomiting, including chemotherapy, in treatment-phase patients with cancer [13-16]. However, additional research into non-pharmacological support for nausea and vomiting in cancer progression is necessary to gain insights into these symptoms among patients with terminal cancer.

Nursing support refers to non-pharmacological support provided by nurses, the healthcare professionals expected to provide the best evidence-based practices. Previous studies have included patients with chemotherapy-induced nausea and vomiting. The Oncology Nursing Society (ONS) guidelines provide information on nursing support for chemotherapy-induced nausea and vomiting [17]. However, because of insufficient evidence on nursing support for nausea and vomiting associated with cancer progression, it is often provided based on nurses’ clinical experience. Hence, we conducted a comprehensive review of research on nursing support for nausea and vomiting, which is not induced by medical therapies, among patients with all stages of cancer. This was done to identify a broad range of nursing support approaches for nausea and vomiting associated with cancer progression.

## Review

Objective

We conducted a scoping review exploring practices currently used by nurses to reduce nausea and vomiting in patients with cancer.

Methodology

To provide an overview of articles on nursing support for patients with cancer, we utilized the method developed by Arksey and O’Malley [18] and conducted a scoping review, in accordance with the Preferred Reporting Items for Systematic Reviews and Meta-analyses (PRISMA) Extension for Scoping Reviews (PRISMA-ScR) reporting guidelines and checklist [19]. In a scoping review, data are pooled from previously conducted research (rather than by conducting a new quality assessment or critical appraisal) following a five-step process: (i) identify the research question; (ii) identify relevant studies; (iii) select studies; (iv) chart the data and major issues; and (v) collate, summarize, and report the results. Our scoping review protocol [20] was published prospectively.

This study is a scoping review of nausea and vomiting, which are symptoms that were addressed in the protocol paper. The definition of nursing support used in this study has been described in the protocol paper. Briefly, it refers to the support that nurses can provide [20]. We, the authors of this review, held discussions among ourselves to determine whether each type of support extracted from the published studies met this standard (could be implemented by nurses caring for patients with advanced cancer) according to the following procedure. First, if the care provider was a nurse, the care was identified as nursing support. Next, if the support provider was not a nurse, the researchers, who were all nurses or physicians, examined the support needs to determine whether nurses could implement the support in their daily clinical practice. Each type of implementable support was identified as a type of nursing support.

Step 1: Identify the Research Question

In the first stage of the scoping review, we identified the key research question: What types of nursing support are provided to reduce the nausea and vomiting experienced by patients with cancer?

Step 2: Identify Relevant Studies

We searched the PubMed, the Cumulative Index to Nursing and Allied Health Literature, Cochrane Central Register of Controlled Trials in the Cochrane Library, and Ichushi-Web of the Japan Medical Abstract Society databases for content published from the inception of each database through July 31, 2023. The original search formulas were created while using PubMed, our initial search source. Subsequent search formulas were created, as needed, to work with the remaining databases (see Appendix 1 of the protocol paper for details) [20]. Two authors (MK and KK) completed the initial search in consultation with the librarian.

Step 3: Select Studies

A scoping review was conducted according to the PRISMA-ScR guidelines. The eligibility criteria were described in the protocol paper [20] and determined by physicians and nurses specializing in symptom management for patients with cancer. Studies were included based on the following eligibility criteria: (i) patients with cancer over 18 years of age, (ii) intervention or observational studies that focused on relieving nausea and vomiting, (iii) nursing support, and (iv) quantitative data showing outcomes. We excluded papers clearly showing that nausea and vomiting were caused by cancer treatment, papers in which over 20% of the participants did not have cancer, papers with secondary analyses, and those published in languages other than Japanese or English. Literature on nausea and vomiting related to treatment was intentionally excluded from this study to reduce the heterogeneity of the target population by focusing on nausea and vomiting caused by advanced cancer disease-related symptoms.

The web-based application Rayyan, a software for systematic reviews that facilitates initial screening through titles and abstracts, was used to analyze the identified articles [21]. The search results were combined, and duplicates were removed. We checked the titles and abstracts of the extracted articles according to the selection criteria. The full text of the extracted articles was then checked according to eligibility criteria; articles that did not meet the criteria were excluded. In accordance with the search strategy, the process of extracting literature was conducted independently by two authors (MK and KK). Disagreements regarding inclusion were resolved by reaching a consensus through discussions between these two authors.

Step 4: Chart the Data and Major Issues

A Microsoft Excel spreadsheet was used to organize the data from the collected articles. The input to the spreadsheet was conducted by two independent authors (MK and KK). A form was created to extract the study characteristics, including the first author, publication year, country, title, study design, sample size, age, type of cancer, and outcome measurement tools. This study also extracted details about the nursing support intervention or support program based on the following components: (i) type of support, (ii) components, (iii) results of the interventions for nausea and vomiting, and (iv) population status (terminal or not).

Step 5: Collate, Summarize, and Report the Results

The authors organized the collected data, classified them by group or summarized them, and reported them. Data were imported into Microsoft Excel for validation and coding and summarized in a spreadsheet.

Results

**Figure 1:**
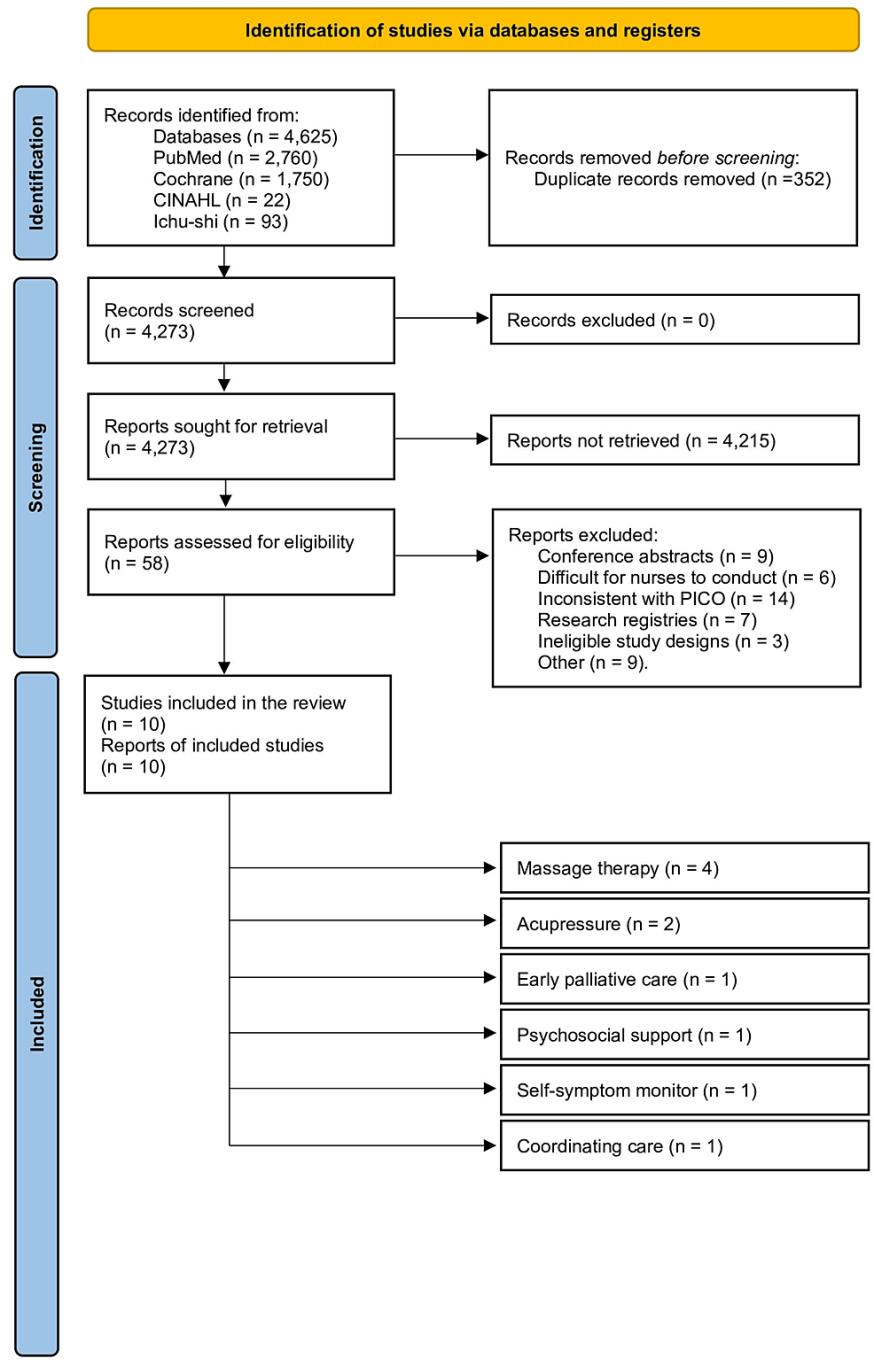
PRISMA flow diagram. PRISMA: Preferred Reporting Items for Systematic Reviews and Meta-Analyses; PICO: Population, Intervention, Comparison, and Outcomes

The literature screening process and results are presented in Figure 1. As a result of the literature search process, 4,625 scientific articles were extracted. After removing the duplicate articles, we reviewed the titles and abstracts of 4,273 articles. This resulted in 58 articles being eligible for full-text review, of which 10 met the selection criteria [22-31]. The agreement rate between the reviewers was 82.8% (48/58).

Summary of Study Characteristics

Table 1 summarizes the characteristics of the selected studies. Based on the types of study design, there were six randomized controlled trials (RCTs), three prospective observational studies, and one before-after study with no controls. The types of cancers included in the articles were colorectal (n = 6), breast (n = 5), lung (n = 5), pancreatic (n = 4), gynecological (n = 3), stomach (n = 3), and sarcoma (n = 3). The total sample size of the study population was 793 patients (range: 12-281) for intervention studies and 4,333 patients (range: 20-4,197) for observational studies. The measures of nausea and vomiting outcomes included the Edmonton Symptom Assessment System Revised (n = 4), Visual Analog Scale (n = 3), Numerical Rating Scale (n = 1), European Organization for Research and Treatment of Cancer Quality of Life Questionnaire Core 30 (n = 1), and Spitzer Quality of Life Index (n = 1).

**Table 1:** Summary of study characteristics.

Author (publication year)	Country	Title	Study design	Study sample size (n)	Participants’ age (years)
Anderson et al. (2021) [22]	USA	Foot reflexology: An intervention for pain and nausea among inpatients with cancer	RCT	40	Range: 18–80
Tsugita et al. (2021) [23]	Japan	High feasibility and safety, but negligible efficacy of acupressure for treating nausea in cancer patients admitted to the palliative care unit: a pilot study	Before-after study	12	Mean (range) = 70 (56–87)
Perkins et al. (2020)[24]	USA	Does acupressure help reduce nausea and vomiting in palliative care patients? A double-blind randomized controlled trial	Observational study	55	Intervention: mean = 65.5; Control: mean = 67.0
Fink et al. (2020) [25]	UK	A quality brief of an oncological multisite massage and acupuncture therapy program to improve cancer-related outcomes	RCT	4197	Unknown
Zimmermann et al. (2019) [26]	Canada	Phase 2 trial of symptom screening with targeted early palliative care (STEP) for patients with advanced cancer	Observational study	116	Intervention: mean (MD) = 61.2 (±12.6); Control: mean (MD) = 62.7 (±11.9)
Wang et al. (2015) [27]	Taiwan	The effect of abdominal massage in reducing malignant ascites symptoms	RCT	80	Total: mean (MD) = 59.11 (35–83)
Arving et al. (2007) [28]	Sweden	Individual psychosocial support for breast cancer patients: a randomized study of nurse versus psychologist interventions and standard care	RCT	179	Intervention: mean (range) = 55 (34–72) years; Control: mean (range) = 55 (25–87)
Hoekstra et al. (2006) [29]	Netherlands	Using the symptom monitor in a randomized controlled trial: the effect on symptom prevalence and severity	RCT	146	Intervention: mean = 64.1; Control: mean = 64.6
Giasson and Bouchard (1998) [30]	Canada	Effect of therapeutic touch on the well-being of persons with terminal cancer	Observational study	20	Total range: 38–68
Addington-Hall et al. (1992) [31]	UK	Randomised controlled trial of effects of coordinating care for terminally ill cancer patients	RCT	281	Range: 18–(upper limit unknown)

Nursing Support Components

Nursing support extracted from the 10 articles was classified into six types: massage therapy (n = 4), acupressure (n = 2), early palliative care (n = 1), psychosocial support (n = 1), self-symptom monitoring (n = 1), and coordinating care (n = 1). This support was delivered by nurses (n = 5), researchers (n = 4), physicians (n = 1), and skill-trained therapists (n = 1). Seven articles included patients with terminal cancer. The types of nursing support provided to terminally ill patients with cancer included massage therapy, acupressure, early palliative care, self-symptom monitoring, and coordinating care. Details of the components of the nursing support interventions and support programs are shown in Table 2.

**Table 2:** Nursing support components for nausea and vomiting.

Type of support	Component	Provider	Includes terminal illness
Massage therapy [22,25,27,30]	Reflexology techniques were adopted from the Ingham method and included thumb and finger walking, hooking in, backing up, and rotating on the point; 15–20 minute/session	Researcher	✕
	Oncology massage consisting of Reiki (levels I & II), aromatherapy, and guided relaxation exercises	Skill-trained therapist	Unknown
	The main massage maneuvers were straight rubbing (back and forth rubbing), point rubbing, and kneading; 15-minute gentle abdominal massage twice daily (at 7–8 a.m. and 4–5 p.m.) for three consecutive days	Nurse	〇
	Used Rogers’s nursing conceptual model, Science of Unitary Human Beings. Non-contact therapeutic touch is a consciously directed process of energy exchange during which the hands are used as a focus to repattern the human energy field; 15–20 minutes/session, three times	Nurse	〇
Acupressure [23,24]	Neiguan and Zusanli were set as the acupressure sites. Acupressure was performed on each limb, with a pressure time of 30 seconds for each acupressure point (three sets at each acupressure point; total intervention time = 6 minutes)	Researcher	〇
	Acupressure on the P6 site with a wristband for three days	Researcher	〇
Early palliative care [26]	A nurse-led telephone triage line was available, with after-hours physician telephone support ensuring 24/7 coverage. If needed, patients were referred for home nursing care (in conjunction with clinic visits)	Physician, Nurse	〇
Psychosocial support [28]	First session: The patient was asked to discuss their disease history. Second session: The patient’s problems were identified; strategies—problem-solving, relaxation and distraction techniques, ways to improve communication, activity scheduling—that could help to manage these problems were taught to the patient	Nurse	✕
Self-symptom monitoring [29]	The intervention consisted of a weekly patient self-assessment of physical symptoms using the symptom monitor	Researcher	〇
Coordinating care [31]	The coordinators were responsible for ensuring that patients received appropriate and well-coordinated services that were tailored to their individual needs and circumstances	Nurse	〇

Discussion

This study identified that nursing support reduces nausea and vomiting experienced by patients with cancer. Based on the findings of the scoping review, we identified six classifications of nursing support approaches to improve nausea and vomiting in patients with cancer.

Massage therapy, defined as therapeutic manipulation using the hands or mechanical devices to maintain the suppleness of the body, was extracted from four of the 10 selected articles. Massage therapy is being increasingly used for symptom relief in patients with cancer [32-34]. The specific content of support is foot reflexology, multimodal care (care that combines Reiki levels, aromatherapy, and guided relaxation exercises), abdominal massage, and therapeutic touch. Further, support methods have been implemented in a wide variety of ways. Two of the four sessions were conducted by nurses, and three lasted approximately 15 minutes. Therefore, massage therapy is most likely implemented using a nurse-led approach. In addition, this study considered it possible to incorporate massage therapy into the usual nursing care practice because it can be provided in a short period. Further, the degree of invasiveness for the patient depends on the intensity of the massage, but nursing support can be adapted regardless of the patient’s situation if implemented according to the patient’s condition and preferences.

Acupressure was extracted from two of the 10 included articles. Specific support was provided to the acupressure sites PC-6 and ST36, both of which were supported by acupressure for several minutes. These sites are commonly used to treat nausea and vomiting. Previous studies have been conducted among patients with cancer experiencing chemotherapy-induced nausea and vomiting [35]. The ONS [17] guidelines regarding acupressure as a support approach for chemotherapy-induced nausea and vomiting are summarized as “effectiveness not established,” as its effectiveness has not been clarified. Contrastingly, the National Comprehensive Cancer Network [36] guidelines recommend acupressure as a type of self-management support for nausea and vomiting. Therefore, acupressure could help reduce nausea and vomiting in patients with cancer.

Early palliative care was extracted from one of the 10 articles. The specific support provided included nurse-led symptom triage and support for symptom management, through multidisciplinary collaboration. Early palliative care has been reported to improve the quality of life of patients with cancer [2,37]. Therefore, in addition to improving the symptoms of nausea and vomiting, various other positive effects can be expected, such as an improved quality of life. Consequently, early palliative care provided by a multidisciplinary team could help reduce nausea and vomiting in patients with cancer.

Psychosocial support was extracted from one of the 10 articles. The specific support provided included (a) an assessment of emotional and social problems and (b) a discussion to resolve the problems. Nausea and vomiting affect psychosocial aspects among patients with cancer [38,39]. These studies posit that physical symptoms and psychosocial aspects are related; the researchers also suggest that psychosocial support can be effective, regardless of the patient’s situation. Therefore, psychosocial support for patients with terminal cancer could help reduce the physical, emotional, and social aspects of their disease-related nausea and vomiting.

Self-symptom monitoring was extracted from one of the 10 articles. The specific support provided was for patients to self-evaluate their symptoms and report the same to their healthcare providers once a week. Monitoring their own symptoms could give patients a sense of control over their symptoms, which could lead to symptom reduction [40,41]. Self-symptom monitoring can lead to the self-management of symptoms and could be broadly helpful for symptoms other than nausea and vomiting. Self-symptom monitoring is also considered an important support tool for self-care, early detection, and the treatment of symptoms.

Coordinating care was extracted from one of the 10 articles. The specific support provided was advice on accessing local social services and promoting community linkages. In addition, in the included study, coordinating care was provided by nurses. Patients could experience worsening symptoms of nausea and vomiting, even when living in a community. Therefore, the role of the care coordinator is important for the continuity of care. In addition, nurses actively provide medical care in various clinical settings such as local communities and hospitals; thus, they may be ideal for coordinating care [42,43]. In this setting, this study considers it important for healthcare providers to collaborate with each other and patients, to coordinate patients with cancer, and to ensure that they receive appropriate social services, which meet their needs to improve symptoms. Coordination could be helpful if a hardware environment is available to provide support.

Seven of the 10 articles included terminally ill patients with cancer as the target population. In addition, five of the six types of nursing support included studies on terminally ill patients with cancer. This study intentionally excluded the literature on nausea and vomiting related to cancer treatment to avoid heterogeneity. Consequently, most of the extracted literature focused on patients with terminal cancer. Therefore, this study provides suggestions for nursing support for terminally ill patients with cancer as well as all cancer progression stages. The degree of invasiveness, including adverse events, is an important factor when considering the potential availability of nursing support for terminally ill patients with cancer [44]. Massage and acupressure have different degrees of invasiveness, depending on the intensity of their use. However, nursing support could be applied for patients with terminal cancer, if implemented according to patients’ conditions and preferences. Early palliative care, psychosocial support, self-symptom monitoring, and coordinating care are not physically invasive; therefore, this could support oncology patients, particularly those with terminal cancer. However, the applicability of these supports has not been verified. Therefore, it is important to evaluate items such as complexity, compatibility, available resources, and access to knowledge/information to determine their applicability to patients with cancer [45].

Finally, the number of RCTs was limited to six articles. Few clinical trials of nausea and vomiting related to cancer progression have been conducted because of the complex and multicausal nature of nausea and vomiting, respondent burden, and ethical issues [46]. Indeed, unlike cancer treatment-derived studies, there is insufficient evidence for nausea and vomiting in relation to cancer progression [47,48]. Thus, future research should examine the support for nausea and vomiting associated with cancer progression. Additionally, five of the 10 articles were published in 2019 or later, suggesting that nursing support for nausea and vomiting could have been the topic of much research attention in recent years. In addition, nine of the 10 articles focused on various cancer types rather than a specific one. Therefore, although the extracted support is versatile, it could be affected by the type of cancer.

This scoping review has several research limitations. First, it only included articles in English and Japanese; hence, some relevant articles published in other languages could have been excluded. Second, it was not designed to evaluate the quality of the studies. Therefore, this conclusion is not based on a synthesis of evidence regarding nursing support for reducing nausea and vomiting. Third, the search was limited to studies in which at least 80% of the participants were patients with cancer; thus, studies conducted primarily on patients without cancer were excluded. The excluded studies could have presented evidence-supporting treatments from non-specialized clinical practice.

## Conclusions

This scoping review comprehensively explored the nursing support provided to reduce nausea and vomiting in patients with cancer. The results of this scoping review classified nursing support for nausea and vomiting in patients with cancer into six types, namely, massage therapy, acupressure, early palliative care, psychosocial support, self-symptom monitoring, and coordinating care. In addition, most of the extracted literature focused on patients with terminal cancer. Therefore, this study provides suggestions for nursing support for terminally ill patients with cancer as well as all cancer progression stages. Future research should examine the feasibility of implementing these types of nursing support for nausea and vomiting in patients with terminal cancer, with a prognosis of months or weeks while also exploring effective prognosis-based nursing support. Finally, the study goal was to map nursing support for nausea and vomiting. Therefore, we did not assess the quality of individual studies or examine the effectiveness of interventions. Future research could benefit from assessing the quality of individual studies and testing the effectiveness of interventions.

## Appendices

**Table 3:** Preferred Reporting Items for Systematic Reviews and Meta-Analyses Extension for Scoping Reviews (PRISMA-ScR) Checklist.

Section	Item	PRISMA-ScR checklist item	Reported on page #	
Title				
Title	1	Identify the report as a scoping review.	Title page	
Abstract				
Structured summary	2	Provide a structured summary that includes (as applicable): background, objectives, eligibility criteria, sources of evidence, charting methods, results, and conclusions that relate to the review questions and objectives.	abstract	
Introduction				
Rationale	3	Describe the rationale for the review in the context of what is already known. Explain why the review questions/objectives lend themselves to a scoping review approach.	P2-3	
Objectives	4	Provide an explicit statement of the questions and objectives being addressed with reference to their key elements (e.g., population or participants, concepts, and context) or other relevant key elements used to conceptualize the review questions and/or objectives.	P3	
Methods				
Protocol and registration	5	Indicate whether a review protocol exists; state if and where it can be accessed (e.g., a Web address); and if available, provide registration information, including the registration number.	P3	
Eligibility criteria	6	Specify characteristics of the sources of evidence used as eligibility criteria (e.g., years considered, language, and publication status), and provide a rationale.	P3	
Information sources*	7	Describe all information sources in the search (e.g., databases with dates of coverage and contact with authors to identify additional sources), as well as the date the most recent search was executed.	P4	
Search	8	Present the full electronic search strategy for at least 1 database, including any limits used, such that it could be repeated.	P4 (protocol paper)	
Selection of sources of evidence†	9	State the process for selecting sources of evidence (i.e., screening and eligibility) included in the scoping review.	P4	
Data charting process‡	10	Describe the methods of charting data from the included sources of evidence (e.g., calibrated forms or forms that have been tested by the team before their use, and whether data charting was done independently or in duplicate) and any processes for obtaining and confirming data from investigators.	P4-5	
Data items	11	List and define all variables for which data were sought and any assumptions and simplifications made.	P4	
Critical appraisal of individual sources of evidence§	12	If done, provide a rationale for conducting a critical appraisal of included sources of evidence; describe the methods used and how this information was used in any data synthesis (if appropriate).	P2-3	
Section	Item	PRISMA-ScR checklist item	Reported on page #	
Synthesis of results	13	Describe the methods of handling and summarizing the data that were charted.	P5	
RESULTS				
Selection of sources of evidence	14	Give numbers of sources of evidence screened, assessed for eligibility, and included in the review, with reasons for exclusions at each stage, ideally using a flow diagram.	Figure 1, P5	
Characteristics of sources of evidence	15	For each source of evidence, present characteristics for which data were charted and provide the citations.	Table 1, P5-6	
Critical appraisal within sources of evidence	16	If done, present data on critical appraisal of included sources of evidence (see item 12).	-	
Results of individual sources of evidence	17	For each included source of evidence, present the relevant data that were charted that relate to the review questions and objectives.	Table 1, P5-6	
Synthesis of results	18	Summarize and/or present the charting results as they relate to the review questions and objectives.	Table 2, P5-6	
Discussion				
Summary of evidence	19	Summarize the main results (including an overview of concepts, themes, and types of evidence available), link to the review questions and objectives, and consider the relevance to key groups.	P6-8	
Limitations	20	Discuss the limitations of the scoping review process.	P8-9	
Conclusions	21	Provide a general interpretation of the results with respect to the review questions and objectives, as well as potential implications and/or next steps.	P9	
Funding				
Funding	22	Describe sources of funding for the included sources of evidence, as well as sources of funding for the scoping review. Describe the role of the funders of the scoping review.	P10	
JBI = Joanna Briggs Institute; PRISMA-ScR = Preferred Reporting Items for Systematic Reviews and Meta-Analyses extension for Scoping Reviews. * Where sources of evidence (see second footnote) are compiled from, such as bibliographic databases, social media platforms, and Web sites. † A more inclusive/heterogeneous term used to account for the different types of evidence or data sources (e.g., quantitative and/or qualitative research, expert opinion, and policy documents) that may be eligible in a scoping review as opposed to only studies. This is not to be confused with information sources (see first footnote). ‡ The frameworks by Arksey and O’Malley (6) and Levac and colleagues (7) and the JBI guidance (4, 5) refer to the process of data extraction in a scoping review as data charting. § The process of systematically examining research evidence to assess its validity, results, and relevance before using it to inform a decision. This term is used for items 12 and 19 instead of "risk of bias" (which is more applicable to systematic reviews of interventions) to include and acknowledge the various sources of evidence that may be used in a scoping review (e.g., quantitative and/or qualitative research, expert opinion, and policy document).				
From: Tricco AC, Lillie E, Zarin W, O'Brien KK, Colquhoun H, Levac D, et al. PRISMA Extension for Scoping Reviews (PRISMAScR): Checklist and Explanation. Ann Intern Med. 2018;169:467–473. doi: 10.7326/M18-0850.				
